# Space Use and Habitat Selection by Resident and Transient Red Wolves (*Canis rufus*)

**DOI:** 10.1371/journal.pone.0167603

**Published:** 2016-12-21

**Authors:** Joseph W. Hinton, Christine Proctor, Marcella J. Kelly, Frank T. van Manen, Michael R. Vaughan, Michael J. Chamberlain

**Affiliations:** 1 Warnell School of Forestry and Natural Resources, University of Georgia, Athens, Georgia, United States of America; 2 Department of Fish and Wildlife Conservation, Virginia Tech, Blacksburg, Virginia, United States of America; 3 U.S. Geological Survey, Northern Rocky Mountain Science Center, Interagency Grizzly Bear Study Team, Bozeman, Montana, United States of America; Sichuan University, CHINA

## Abstract

Recovery of large carnivores remains a challenge because complex spatial dynamics that facilitate population persistence are poorly understood. In particular, recovery of the critically endangered red wolf (*Canis rufus*) has been challenging because of its vulnerability to extinction via human-caused mortality and hybridization with coyotes (*Canis latrans*). Therefore, understanding red wolf space use and habitat selection is important to assist recovery because key aspects of wolf ecology such as interspecific competition, foraging, and habitat selection are well-known to influence population dynamics and persistence. During 2009–2011, we used global positioning system (GPS) radio-telemetry to quantify space use and 3^rd^-order habitat selection for resident and transient red wolves on the Albemarle Peninsula of eastern North Carolina. The Albemarle Peninsula was a predominantly agricultural landscape in which red wolves maintained spatially stable home ranges that varied between 25 km^2^ and 190 km^2^. Conversely, transient red wolves did not maintain home ranges and traversed areas between 122 km^2^ and 681 km^2^. Space use by transient red wolves was not spatially stable and exhibited shifting patterns until residency was achieved by individual wolves. Habitat selection was similar between resident and transient red wolves in which agricultural habitats were selected over forested habitats. However, transients showed stronger selection for edges and roads than resident red wolves. Behaviors of transient wolves are rarely reported in studies of space use and habitat selection because of technological limitations to observed extensive space use and because they do not contribute reproductively to populations. Transients in our study comprised displaced red wolves and younger dispersers that competed for limited space and mating opportunities. Therefore, our results suggest that transiency is likely an important life-history strategy for red wolves that facilitates metapopulation dynamics through short- and long-distance movements and eventual replacement of breeding residents lost to mortality.

## Introduction

Reintroduced populations of large carnivores are frequently re-exposed to anthropogenic factors originally responsible for their extirpation, and limited knowledge hinders developing effective management responses to reduce extinction risk and promote conditions for recovery towards a self-sustaining population [1–4]. Consequently, recovery of carnivore populations remains a challenge in which insufficient area requirements, negative public perception, and policies designed to favor livestock or other wildlife species continue to hamper recovery efforts [4–6]. Complex spatial dynamics and group living that facilitate population persistence of large carnivores are often poorly understood, underappreciated, or simply neglected by wildlife managers and ecologists [1,2,4,7]. For example, wolves maximize reproductive fitness through group living and by defending year-round territories in which pack size influences success in territorial defense [8–12]. Human-caused killings of essential members (e.g., breeders) in wolf packs are problematic to recovery efforts because they destabilize social structures and increase reproductive failure [3,11–16]. For eastern wolves (*Canis lycaon*) and red wolves (*Canis rufus*), increased rates of human-caused mortality resulting in collapsed territories facilitates coyote (*Canis latrans*) encroachment and increased rates of hybridization between coyotes and wolves [3,14,16–18]. Consequently, recovery of the critically endangered red wolf has been challenging because of its vulnerability to extinction via anthropogenic mortality and hybridization with coyotes [3,16,19–23].

Indigenous to the eastern United States, red wolves were completely extirpated from their historic range by 1980 and listed as endangered by the United State Fish and Wildlife Service (USFWS), and later reintroduced into eastern North Carolina and the Great Smoky Mountains National Park during 1987 and 1991, respectively [19,22,24]. Although the North Carolina reintroduction continues, reintroduction to the Great Smoky Mountains National Park was terminated after 6 years because red wolves were unable to maintain territories within park boundaries while the population experienced low pup survival [25–26]. Likewise, red wolves in eastern North Carolina reside proximate to agricultural habitats on private and federal lands over large, contiguous forested habitats available on USFWS national wildlife refuges [27–30]. Consequently, the USFWS Red Wolf Recovery Program (hereafter Recovery Program) faced potential issues with predicting how the red wolf population would distribute itself on the landscape as it expanded, anticipating logistic and social constraints (i.e., conflict with landowners and hunters), and understanding interactions between red wolves and coyotes.

Previous studies of red wolf space use and habitat selection reported home ranges that varied between 10–150 km^2^, comprising mostly agricultural habitats [27–30]. However, past studies only focused on space use patterns of resident red wolves, did not analyze space use and habitat selection together, and suffered from small sample sizes. For instance, Hinton and Chamberlain [27] only assessed home-range sizes and habitat use by 2 red wolf packs during pup rearing, whereas Chadwick et al. [28] observed yearly home ranges of 4 male wolves. Moreover, Dellinger et al. [29] only measured habitat selection by resident red wolves and Karlin et al. [30] measured habitat selection using data obtained from VHF radio-collars collected during diurnal (0900–1200 hours) telemetry flights. Understanding space use and habitat selection patterns of resident red wolves is important because residents comprise the reproductive portion of the population. However, the spatial dynamics of the reintroduced population consists of resident red wolves that have mates and maintain packs and transient wolves that are solitary individuals seeking territories and mating opportunities. Therefore, our current knowledge of red wolf space use patterns is incomplete because of the exclusion of transient red wolves from previous studies. Indeed, transient individuals are known to exist in coyote [31–33], eastern wolf [17], and gray wolf [34–37] populations, but their ability to traverse expansive areas makes transients difficult to monitor and study without global positioning system (GPS) and satellite technology [33]. Despite these difficulties, transiency is recognized to have a functional role in the population dynamics of wolves through emigration and replacement of breeding residents lost to mortality [3,12–13,15,36].

Hinton et al. [33] reported space use and habitat selection of coyotes sympatric with red wolves in eastern North Carolina. Like wolves, transient coyotes were observed replacing breeding residents lost to mortality [33]. Consequently, Hinton et al. [33] suggested that transiency was an important life-history strategy facilitating coyote metapopulation dynamics through dispersal and replacement of resident breeders. Indeed, Hinton et al. [3] reported increased occurrence of coyote encroachment and replacement of resident red wolves after resident wolf breeders were killed by humans. Although red wolves exhibit assortative mating patterns [16,38], coyotes were capable of pair-bonding with surviving red wolves when no transient wolves were available to encroach into territories that were disrupted by anthropogenic mortality [3]. Consequently, pair-bonding with coyotes by surviving red wolf residents formed congeneric breeding pairs that hybridized [3,22,33]. Therefore, the ability of red wolves to recover breeding territories is likely a function of the number of transients traversing the landscape seeking mating opportunities, in which having fewer wolves in the Red Wolf Recovery Area created opportunities for coyotes replace wolves lost to human-caused mortality through encroachment [3].

Our understanding of how the reintroduced red wolf population in eastern North Carolina has distributed itself on the landscape and used available habitat remained incomplete because studies examining ecology of red wolf space use were limited. To improve our understanding of red wolf spatial requirements, we investigated patterns of space use and habitat selection by resident and transient wolves and examined their implications for red wolf recovery. To accomplish this, we quantified size of areas used by resident and transient red wolves and described habitats comprising those areas. We then compared differences in habitat selection by resident and transient red wolves by developing resource-selection functions (RSFs) to predict and map the relative probability of habitat use by wolves.

## Materials and Methods

### Study area

**Fig 1:**
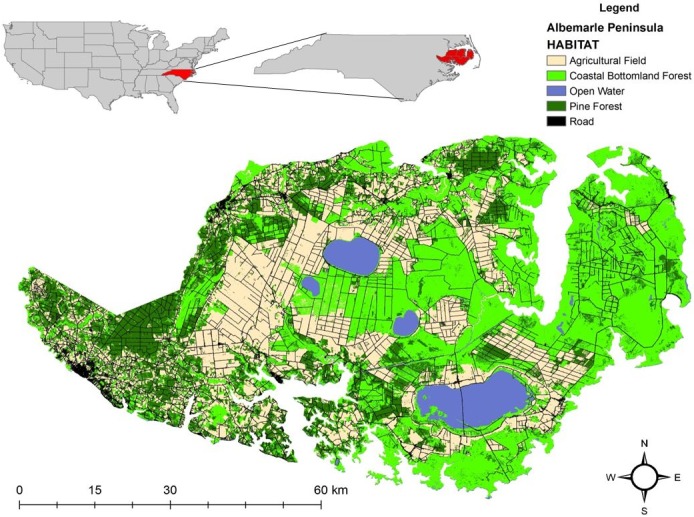
Map of the Albemarle Peninsula of northeastern North Carolina with primary habitat types during 2009–2011.

Our study area was located on Albemarle Peninsula in eastern North Carolina where recovery of the reintroduced red wolf population occurred (Fig 1). The Red Wolf Recovery Area (hereafter Recovery Area) was approximately 6,000 km^2^ of federal, state, and private lands and included 5 counties (Beaufort, Dare, Hyde, Tyrrell, and Washington). The landscape of the Recovery Area comprised a row-crop agricultural-bottomland forest matrix (Fig 1). Agricultural crops (i.e., corn, cotton, soybean, winter wheat) comprised approximately 30% of the vegetative cover during spring and summer. After crops were harvested during early fall, agricultural fields were barren through fall and winter. Managed pine (*Pinus* spp.) forests comprised approximately 15% of vegetative cover. Other prominent vegetative cover types were coastal bottomland forests and pocosin (35%), herbaceous wetlands and saltwater marshes (5%), and other minor vegetative communities (10%). Approximately 5% of the Recovery Area comprised open water such as lakes, rivers, and streams. Variability in elevation on the Albemarle Peninsula was minor and ranged between 0–50 m. The climate was representative of the mid-Atlantic comprising 4 full seasons (spring, summer, fall, and winter), nearly equal in length with annual averages in precipitation ranging between 122 to 132 cm. Summers were hot and humid with temperatures ranging between 27°C to 38°C whereas winters were relatively cool with temperatures ranging between -4°C to 7°C.

#### Capture and monitoring

The USFWS Recovery Program conducted annual trapping to capture and radio-mark juvenile and adult red wolves for long-term monitoring and management of the reintroduced population [23,39–40]. We assisted annual trapping efforts during 2009–2011 to deploy GPS radio collars on red wolves. During this period, the Recovery Program annually monitored 83–94 radio-collared red wolves in 16–17 packs [3,23,40]. Each year, 10–15 radio-collared red wolves not associated with known packs or breeding pairs were assumed to be transient (e.g., dispersing) [41]. The red wolf is listed as critically endangered under the United States Endangered Species Act (ESA) and by the International Union for Conservation of Nature (IUCN). Therefore, we operated under a cooperative agreement with the USFWS Recovery Program to trap and handle red wolves. Our methods to capture, handle, and process red wolves were in cooperation and concordance with the USFWS, approved by the Louisiana State University Agricultural Institutional Animal Care and Use Committee (Protocol Number AE2009-19) and met guidelines recommended by the American Society of Mammalogists [42]. Permission to access private lands for trapping occurred under memorandum of agreements (MOAs) between individual landowners and the Recovery Program and, when MOAs did not exist, we received permission to trap from landowners.

During 2009–2011, we captured red wolves using padded foot-hold traps (Victor no. 3 Softcatch, Lititz, Pennsylvania, USA) from October through May. Red wolves were typically restrained using a catchpole, muzzle, and hobbles. Although most red wolves were not anesthetized, some were chemically immobilized with an intramuscular injection of ketamine HCl and xylazine HCl to inspect inside the mouth for injuries. Red wolves were sexed, measured, and weighed. Ages and genetic confirmation of captured red wolves were known if individuals were carrying a subcutaneous passive integrated transponder (PIT) tags inserted into the animal during annual surveys of suspected red wolf dens [23,39,43]. Ages of individuals without PIT tags were estimated by tooth wear [44–45], and a blood sample was taken for genetic identification. We categorized red wolves ≥2 years old as adults, <2 but ≥1-year-old as juveniles, and <1-year-old as pups. Prior to release at the original capture sites, we fitted adult and juvenile red wolves with a mortality-sensitive GPS radio-collar (Lotek 4400S, Newmarket, Ontario, Canada) that recorded a location every 5 hours on a 24-hour rotating schedule throughout the year.

The Recovery Program monitored radio-collared red wolves 2 times a week from aircraft to identify and monitor territories in the Recovery Area [23]. We identified resident pairs of red wolves as radio-collared individuals of breeding age (≥2 years old) that were temporally and spatially associated with one another and defending a territory for ≥4 months as a breeding pair [3,33]. Recovery Program biologists confirmed breeding pair status during spring den work by locating dens and daybeds of radio-marked females to verify and obtain pup counts for each radio-collared breeding pair [23,39–40,46–47]. We also classified non-dispersing juveniles fitted with radio-collars as residents. To reduce autocorrelation, we only used one GPS radio-collared red wolf from a known pack and only used non-dispersing juveniles when resident breeding pairs were not fit with GPS radio collars. We classified radio-collared red wolves as transients when they were solitary and not associated with other radio-collared individuals and displayed nomadic, wandering movements throughout the Recovery Area.

### Data analysis

We used dynamic Brownian bridge movement models (dBBMM) to estimate space use of resident and transient red wolves [48–49]. We used dBBMMs from the R package ‘moveud’ [50] in Program R [51] to estimate utilization distributions (UD) along the full movement track of each red wolf. The dBBMM is a continuous-time stochastic movement model that incorporates time and distance, location error, and an estimate of the Brownian motion variance ($\sigma_{m}^{2}$ between 2 successive locations) [52–53]. The dBBMM identifies changes in movement speed and direction using a sliding window along the movement path to calculate a $\sigma_{m}^{2}$ for each time step that correspond to different movement patterns, which are then averaged to produce a final, independent $\sigma_{m}^{2}$ for each path step [48–49,52,54]. For full tracks of each wolf, we used a window size of 3 locations (equivalent to 15 hours) and chose a margin of 3 locations on each end of the window in which no breaks in sequential points could occur. Because red wolves are primarily nocturnal, we chose a window size equivalent to 15 hours to account for temporal resolution for each wolf track. Because many factors influence telemetry error and recent studies suggested telemetry error for GPS radio-collars ranged between 10 and 30 m [55], we used an error estimate of 20 m for all locations. We defined 95% and 50% contour intervals as composite home ranges and core areas, respectively. However, resident red wolves established and defended territories whereas transient did not because they were traversing the landscape seeking mates to establish and defend territories with. Therefore, we did not refer to transient space use as home ranges and core areas. Instead, we considered 95% and 50% contour intervals for transients as composite transient ranges and biding areas, respectively [33,56]. To calculate temporal changes in home-range size, we divided each year into 2 6-month seasons based on agricultural activity: growing (1 March–31 August) and harvest (1 September–28 February). These seasons reflected the strong anthropogenic effects of agricultural practices on the landscape. The growing season represented a time when agricultural fields provided cover to red wolves, whereas the harvest season represented a time when those fields were completely barren, and presumably offered no cover. Additionally, the harvest season corresponded with the red wolf and coyote dispersal and breeding season whereas the growing season corresponded with the whelping and pup rearing season. The assumptions of normality and homogeneity of variances were violated for home-range size and were corrected by using natural log transformations. We used *t*-tests to examine differences in the size of space maintained by wolves between growing and harvest seasons. We used an alpha value of 0.05 to test for statistical significance.

We estimated predominant types of vegetative cover in the Recovery Area using a 30-m resolution digital landscape map of vegetative communities developed by the North Carolina Gap Analysis [57]. For the habitat analysis, we reduced vegetative cover into 4 general habitat classes: agriculture, coastal bottomland forest, pine forest, and wetlands (e.g., herbaceous wetlands, marshes, and pocosin). We developed layers for roads and habitat edges (hereafter edges) because previous studies reported use of roads and edges by wolves for travel and foraging opportunities [58–60]. We created distance raster layers for habitat classes, roads and edges by using the Euclidean Distance tool in the Spatial Analyst toolbox in ArcGIS 10.1 (Environmental Systems Research Institute Inc., Redlands, California) to calculate distances from every 30-m pixel the closest landscape features [33,60–61]. To determine if proportional habitat cover differed among home ranges, core areas, transient ranges, and biding areas, we used analysis of variance (ANOVA) and Tukey tests for multiple comparisons. We also used *t*-tests to compare mean body mass and age of resident and transient red wolves. We used an alpha value of 0.05 for all tests of significance. The assumptions of normality and homogeneity of variances were checked by visual inspection of residual plots; the assumptions were met. Assumptions of independence were violated for statistical tests when individual red wolves changed residency and transiency statuses. To maintain independence, we only included transient data from red wolves with both statuses in our analyses.

We followed the Design III (3^rd^-order selection) approach suggested by Manly et al. [62] to examine the relationship between habitats and red wolf space use within their home ranges and transient ranges. Individual red wolves were our replicates and resource availability was measured for each wolf using random locations within home ranges and transient ranges. We used a binomial approach to estimate RSFs within home ranges and transient ranges using distance-based landscape variables (habitat classes, roads, and edges). We inferred selection when known (used) locations were closer to landscape variables than were random (available) locations. We inferred avoidance when known locations were farther from landscape variables than random locations. We developed RSF models for red wolves with Bayesian generalized linear mixed models (GLMMs) using the R package ‘MCMCglmm’ v. 2.22.1 [63] with a binary (0 = available, 1 = used) response variable. We included random intercepts for individual red wolves in each model to account for individual variation and unequal number of telemetry locations per wolf. Prior to modeling, we rescaled values for all distance-based variables by subtracting their mean and divided by 2 standard deviations [64].

To develop RSFs for resident and transient red wolves, we used 4 general hypotheses to design 5 *a priori* candidate models to test factors associated with red wolf use of landscape variables. First, we assumed forested habitats provided cover and shelter for red wolves. Second, we assumed linear features, such as edges and roads, provided movement corridors for red wolves. Third, we assumed open, treeless habitats, such as agricultural fields, were preferred hunting areas for red wolves. Finally, we assumed habitats periodically experiencing inundation events, such as wetlands, were avoided by red wolves. We then removed variables associated with each hypothesis from a global model to compare performance of varying model sets to the global model. This allowed us to deduce which variables had the strongest effect on improving model performance. We used deviance information criteria (DIC), a Bayesian analogue to the Akaike’s information criteria, to compare the fit of 5 models of varying complexity [60,65]. We calculated RSFs to develop probability maps of habitat use by resident and transient red wolves in 2 steps. First, we determined if resident and transient behaviors affected resource selection in red wolves when we included all resident and transient locations from our telemetry data, included main effects for all predictor variables, and allowed interactions between a binary variable for red wolf status (resident = 1, transient = 0) and each landscape variable. Second, we partitioned resident and transient locations to develop separate, 3^rd^-order selection coefficients for each landscape feature without interactions. We then evaluated which landscape features were most important and developed probability of habitat use maps for resident and transient coyotes. Because correlation between individual predictor variables were low or modest (all *r* < 48%), our global model sets represented all 6 landscape features (4 habitat classes, roads, and edges). We then used a *k*-fold cross-validation using 10 folds (*k* = 10) to estimate performance of resident and transient RSF models. We assessed selection or avoidance of resource variables using 95% highest posterior density (HPD) credible intervals derived from MCMC simulation to identify fixed-effect beta coefficients that differed from 0.

## Results

During 2009–2011, we monitored 35 red wolves fitted with GPS radio-collars. At time of capture, 29 red wolves were known residents and 6 were known transients; 9 wolves changed status during the study. As a result, we acquired space and habitat selection data from 32 residents and 11 transients. Of the 11 transients, 5 were residents that became transients whereas 4 were transients that became residents. Two transients did not change status during the study. Mean body mass and age of red wolves monitored were 27.1 kg ± 0.4 and 3.1 years ± 0.2, respectively, in which mass (*t*_*29*_ = 0.181, *P* = 0.857) and age (*t*_*29*_ = 0.458, *P* = 0.650) of residents did not differ from transients (Table 1). Home ranges (25.0 km^2^–190.0 km^2^; *t*_*65*_ = 0.461, *P* = 0.647) and core areas (1.9 km^2^–30.5 km^2^; *t*_*65*_ = 0.062, *P* = 0.951) of residents did not differ between seasons (Table 1). We also detected no seasonal difference in transient ranges (122.3 km^2^–680.8 km^2^; *t*_*12*_ = 0.157, *P* = 0.878) and biding areas (8.9 km^2^–120.0 km^2^; *t*_*12*_ = 0.113, *P* = 0.912; Table 1).

**Table 1:** Mean (± SE) body mass, age, and space use of resident and transient red wolves in northeastern North Carolina during 2009–2011.

			Size of area used (km^2^)					
Red wolf status	Mean mass (kg)	Mean age (yr)	Growing^1^		Harvest^2^		Composite^3^	
			95%^4^	50%^5^	95%	50%	95%	50%
Resident	27.2 ± 0.5	3.0 ± 0.2	73.3 ± 8.5	9.1 ± 1.4	67.8 ± 8.3	9.0 ± 1.6	68.4 ± 7.5	8.7 ± 1.3
Transient	26.8 ± 0.8	3.5 ± 0.4	277.9 ± 80.7	27.3 ± 14.5	260.7 ± 66.1	29.3 ± 8.6	319.2 ± 57.3	32.8 ± 10.8

^1^Growing season space use was defined as areas used during March through August.

^2^Harvest season space use was defined as areas used during September through February.

^3^Composite space use was defined as the total area used.

^4^95% probability contour calculated from dynamic Brownian bridge movement models used to estimate the sizes of resident home ranges and transient ranges.

^5^50% probability contour calculated from dynamic Brownian bridge movement models used to estimate the sizes of resident core areas and transient biding areas.

**Fig 2:**
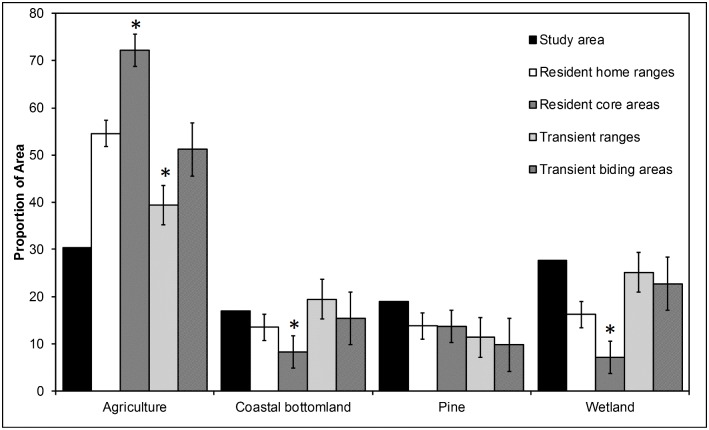
Habitat availability and habitat proportions of space used by resident and transient red wolves in northeastern North Carolina during 2009–2011. Asterisks above the bars represent statistical differences among areas within habitat classes (*P* < 0.05, Tukey’s test). Study area proportions are shown for reference.

Space used by red wolves was comprised mostly of agricultural fields, coastal bottomland forests, pine forests and wetlands (Fig 2). Although we detected no differences in the proportion of pine forests in resident home ranges, core areas, transient ranges, and transient biding areas (*F*_3, 68_ = 0.336, *P* = 0.799), we did detect difference in the proportion of agriculture, coastal bottomland forests, and wetlands (Fig 2). First, agriculture occurred proportionally more in resident core areas than resident home ranges, transient ranges, and transient biding areas (*F*_3, 68_ = 6.034, *P* ≤ 0.001). Second, coastal bottomland forests occurred proportionally more in transient ranges than resident home ranges, resident core areas, and transient biding areas, whereas resident core areas were comprised of proportionally less coastal bottomland forest than resident home ranges, transient ranges, and transient biding areas (*F*_3, 68_ = 2.882, *P* = 0.042). Finally, resident core areas contained proportionally less wetlands than resident home ranges, transient ranges, and transient biding areas (Fig 2; *F*_3, 68_ = 4.737, *P* = 0.005).

**Fig 3:**
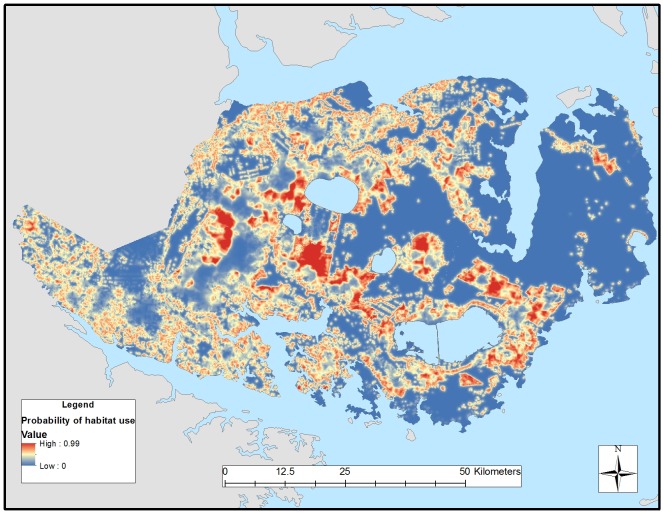
Relative probability of 3^rd^-order habitat selection by resident red wolves across the Albemarle Peninsula in eastern North Carolina during 2009–2011.

**Fig 4:**
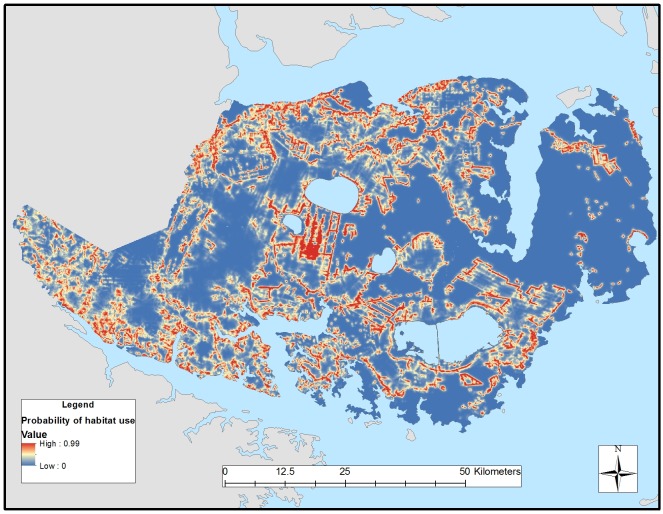
Relative probability of 3^rd^-order habitat selection by transient red wolves across the Albemarle Peninsula in northeastern North Carolina during 2009–2011.

We detected differences in habitat selection between resident and transient red wolves, providing support that behaviors associated with residency and transiency affected resource selection (Tables 2 and 3). Global models best explained 3^rd^-order selection for resident and transient red wolves (Table 4). Except for pine forest, all other covariates were important predictors of transient occurrence in which transients selected agriculture, wetlands, edges, and roads, and avoided coastal bottomland forests (Table 5). Except for edges, all other covariates were important predictors of resident occurrence in which residents selected agriculture, pine forest, wetlands, and roads. Compared with transients, resident red wolves showed greater selection for pine forests and lower selection for edges and roads (Table 5). Our model validation correctly classified 82% and 81% of resident and transient locations, respectively. Differences in habitat selection between residents and transients revealed substantial spatial heterogeneity in probabilities of habitat use in the Recovery Area (Figs 3 and 4) despite their general affinities for similar vegetative cover.

**Table 2:** Comparison of model fit among the null model (no landscape features), and models with and without interactions of status (1 = resident, 0 = transient), used to test hypotheses about red wolf 3^rd^-order resource selection in eastern North Carolina, 2009–2011.

Models	*k*	DIC	ΔDIC	Conclusions
Interactions (status × each landscape feature^1^)	14	170 700	0.00	Interactions strongly supported
No interactions (landscape features only)	8	171 359	659	
Null	2	176 973	6 273	

Shown are deviance information criteria values (DIC), differences between DIC of a given model, and the conclusion regarding support for the interaction term.

^1^ Distance to agriculture, pine forest, wetlands, coastal bottomland forest, agriculture-forest edge, and roads.

**Table 3:** Summary of results from mixed-effect Bayesian resource selection model with interaction of status (resident = 1, transient = 0) for red wolves in eastern North Carolina during 2009–2011.

Model variables	β	95% HPD
Intercept	**-1.367**	-1.481, -1.270
Agriculture	**-0.362**	-0.399, -0.323
Coastal bottomland forest	**0.140**	0.054, 0.218
Pine	0.033	-0.024, 0.086
Wetland	**-0.223**	-0.297, -0.148
Edge	**-0.407**	-0.494, -0.323
Road	**-0.231**	-0.275, -0.187
Agriculture × status	**-0.466**	-0.518, -0.415
Coastal bottomland forest × status	**0.138**	0.054, 0.223
Pine × status	**-0.202**	-0.264, 0.137
Wetland × status	0.092	0.019, 0.174
Edge × status	**0.391**	0.288, 0.476
Road × status	**0.155**	0.106, 0.201

Shown are β coefficients with lower and upper 95% highest posterior density (HPD) credible intervals. Significant effects show in bold. Coefficients of the interaction terms reflect those of resident red wolves relative to the transient red wolves. All variables were based on distance to each landscape feature (i.e., negative values for β indicate closer proximity of red wolf locations to a landscape feature compared with random locations, thus representing selection for that feature).

**Table 4:** Summary of mixed-effect Bayesian resource selection models for predicting red wolf habitat use based on 5 candidate models corresponding to different hypotheses of landscape features potentially affecting 3^rd^-order habitat selection by transient and resident red wolves in northeastern North Carolina, 2009–2011.

Status	Model^1^	*k*	DIC	ΔDIC
Transient	Global model (AG+CB+ED+PI+RD+WL)	8	20 817	0
	No forests (AG+ED+RD+WL)	6	20 827	10
	No wetlands (AG+CB+ED +PI +RD)	7	20 845	28
	No linear features (AG+CB+PI+WL)	6	21 006	189
	No agriculture (CB+ED+PI +RD+WL)	7	21 234	417
Resident	Global model (AG+CB+ED+PI+RD+WL)	8	149 870	0
	No linear features (AG+CB+PI+WL)	6	149 935	65
	No wetlands (AG+CB+ED+PI +RD)	7	149 974	104
	No forests (AG+ED+RD+WL)	6	150 541	671
	No agriculture (CB+ED+PI+RD+WL)	7	153 002	3132

Shown are deviance information criteria values (DIC) and differences between DIC of a given model and the strongest supported model (ΔDIC) for each model considered.

^1^ AG = agriculture, CB = coastal bottomland forest, ED = agriculture-forest edge, PI = pine forest, RD = roads, WL = wetlands

**Table 5:** Parameter estimates from mixed-effect Bayesian resource selection models for transient and resident red wolves in eastern North Carolina during 2009–2011.

Status	Model variables	β	95% HPD
Transient	Intercept	**-1.283**	-1.529, -1.025
	Agriculture	**-0.653**	-0.725, -0.582
	Coastal bottomland forest	**0.114**	0.052, 0.180
	Pine	0.037	-0.030, 0.096
	Wetland	**-0.190**	-0.254, -0.119
	Edge	**-0.391**	-0.476, 0.319
	Road	**-0.322**	-0.388, -0.256
Resident	Intercept	**-1.405**	-1.508, -1.302
	Agriculture	**-0.662**	-0.686, -0.638
	Coastal bottomland forest	**0.284**	0.262, 0.307
	Pine	**-0.165**	-0.194, -0.130
	Wetland	**-0.134**	-0.134, -0.161
	Edge	-0.017	-0.049, 0.017
	Road	**-0.071**	-0.088, -0.053

Shown are β coefficients for the global models (Table 4) with lower and upper 95% highest posterior density (HPD) credible intervals. Significant effects show in bold. All variables were based on distance to each landscape feature (i.e., negative values for β indicate closer proximity of red wolf locations to a landscape feature compared with random locations, thus representing selection for that feature).

## Discussion

The ability of red wolves to persist in eastern North Carolina will depend in part on space use patterns that permit them to navigate and adapt to diverse and dynamic environments, in which population dynamics is facilitated by resident and transient individuals through competition for space and mates [3,12–13,15,36]. Similar to the sympatric coyote population [33], transients comprised a significant proportion of the red wolf population (approximately 30%). Body measurements of red wolves sampled for this study were consistent with body measurements reported in Hinton and Chamberlain [43] and we found no age and size differences between transients and residents, likely because transients consisted of young dispersing and older displaced red wolves. Many red wolves disperse between 1 and 2 years of age [41]. However, resident wolves lose territories when breeding pairs and packs are disrupted by natural (i.e., disease and intraspecific strife) and anthropogenic (i.e., vehicle collisions and shooting deaths) causes [3,23]. Hence, older displaced red wolves also competed with young dispersing wolves for new mates and territories. For instance, we observed 3 instances in which resident red wolf breeders were displaced from established territories and became transients after their mates died from anthropogenic factors. Two of these residents reestablished territories elsewhere, whereas 1 died during transiency. Furthermore, 2 red wolves abandoned their territories and became transient after contracting sarcoptic mange (*Sarcoptes* spp.), and both wolves died during transiency.

Home ranges of resident red wolves were spatially stable, did not vary between seasons, and ranged between 25.0 and 190.0 km^2^. Because many resident red wolves dispersed from natal territories or were displaced from breeding territories, they were likely aware of temporal changes in the environment prior to establishing residency, and acquired sufficient space to accommodate seasonally varying needs and resource availability. Although red wolves had a significantly larger mean home-range size (68.4 km^2^) than did sympatric coyotes (27.2 km^2^) [33], 3 GPS-collared red wolves maintained smaller home ranges (28.0, 35.7, and 55.4 km^2^) while paired with coyotes. Hinton et al. [33] reported a maximum home-range size of 47 km^2^ for coyotes in the Recovery Area. Of the 32 red wolf home ranges observed, 9 (28%) were smaller than 47 km^2^. Comparative studies have reported that carnivore home-range size scales positively with body mass and is likely driven by metabolic costs [66–68]. Indeed, red wolves in congeneric pairs were reported to be predominantly female, and were physically smaller and defended smaller home-ranges than red wolves in conspecific pairings [3,69]. Hinton [69–70] suggested as red wolves and coyotes approached each other in body size, similar use of prey and space may reduce behavioral incompatibilities between consorting individuals and permit the successful formation of congeneric pairs responsible for creating red wolf/coyote hybrids. Because hybridization is a primary threat to the conservation and persistence of eastern wolves [14,18,71–72] and red wolves [3,16,38,21–22,73], we suggest further studies are needed to better understand behaviors and conditions that allow individuals of *Canis* populations to successfully form congeneric breeding pairs responsible for hybridization.

Space used by transient red wolves were unstable, wide-ranging (122.3–680.8 km^2^) and exhibited shifting patterns. However, transients routinely exhibited localized movements (i.e., clusters of locations) for several weeks that averaged 32.8 km^2^, and those areas appeared analogous to small home ranges in both size and habitat composition. Previously, we observed similar space use by transient coyotes and referred to them as biding areas [33,56]. Likewise, we also suggest this behavior may provide benefits to the red wolf population because it increases survivorship of transients via familiarity of areas they roam and, when opportunities arise, they replace residents that die. For example, 6 transient red wolves monitored in this study replaced resident red wolves and coyotes that were killed during the study. Indeed, previous work on gray wolves and red wolves suggested older individuals disperse shorter distances because of their familiarity with the local area and ability to perceive local opportunities [11,41]. Although territorial behavior prevents transients from reproducing, transiency is likely an important trait that allows red wolf populations to recover space and breeding opportunities after suffering mortality events on the landscape. This may be particularly important for these populations to avoid local extinction and persist through metapopulation dynamics [74–76].

Like previous studies, red wolf space use was positively associated with agricultural habitats [27–30]. We documented red wolves establishing home ranges on the edges of agricultural fields and forests with the interior (i.e., core areas) comprising proportionally more agriculture than forest and wetland habitats. Although agricultural crops (i.e., winter wheat and corn) were favored by red wolves as diurnal cover during the growing season (spring through summer), crops harvested by early autumn left agricultural fields barren during the harvest seasons (autumn through winter). When agricultural crops were harvested, red wolves took refuge in forest habitats within 50–300m of edges to barren agricultural fields and roads. After winter planting and when winter wheat reached heights of approximately 0.5 m during the growing season, red wolves abandoned forest habitats and took cover during diurnal hours in wheat fields [27–28]. As winter wheat was harvested during late spring (May and June) and planted to cotton and soybean, red wolves shifted to corn fields. Proportional habitat cover of home ranges and transient ranges of red wolves was similar because residents and transients showed similar selection for agriculture, wetlands, and roads and avoided coastal bottomland forests. However, patterns of habitat selection differed in which resident red wolves had stronger avoidance of coastal bottomland forests than transients and selected pine forests. Transient red wolves strongly selected for edges and roads. Consequently, resident red wolves used agricultural habitats with forest edges to establish territories, whereas transient red wolves concentrated their movements and biding areas proximate to those same habitats via road networks and edges (Figs 3 and 4).

Selection for edges and roads was a primary difference in habitat selection between resident and transient red wolves. Similarly, Hinton et al. [33] also observed sympatric coyotes exhibiting this pattern in the Recovery Area, in which transients favored roads and edges more than residents. We suggest the use of roads by transient red wolves may be related to 2 important aspects of red wolf space use. First, roads may improve the efficiency of transient movements by reducing energetic costs related to shifting and extensive space use that often involve maneuvering through habitats that are inundated or densely vegetated. Additionally, roads are typically associated with edge habitats and could improve foraging opportunities to transients that are excluded from productive habitats found within resident territories. Second, previous studies suggested roads and linear corridors may enhance line of sight and olfactory senses of wolves [58–59]. Pheromones are widely used by animals to initiate passive and indirect interactions and avoid costly physical antagonistic ones [77]. Indeed, scent marking is a fundamental behavior of resident wolves and coyotes to delineate and communicate territory boundaries and avoid antagonistic interactions [78–80]. In addition to facilitating efficient movements, we believe roads and edges improve detection of occupied and vacant areas by transient red wolves allowing them to avoid antagonistic interactions with resident packs. However, wolves are exposed to greater risks of human-caused mortality when using roads [60] and this relationship needs further assessment for red wolves.

Ideally, when the death of red wolf breeders creates vacancies on the landscape, transient red wolves or non-breeding individuals from neighboring packs would acquire vacant territories. Because red wolf and coyote packs maintain exclusive territories, resident red wolves are capable of excluding coyotes from areas they occupy [21]. Indeed, Gese and Terletzky [21] reported that all displacement of canids was unidirectional with larger red wolves displacing smaller coyotes and hybrids and not vice versa. The goal of recovery efforts was to ensure that all *Canis* breeding pairs within the Recovery Area were red wolves [22]. To accomplish this, the Red Wolf Adaptive Management Plan was implemented to minimize hybridization by monitoring red wolf and coyote breeding pairs throughout the Recovery Area, and replacing coyotes and hybrids with red wolves until the Albemarle Peninsula was saturated with red wolf packs [20–22]. In this context, the presence and space use of transients has a profound effect on recovery of red wolves via the ability of transients to replace lost residents and deter coyote encroachment in the Recovery Area.

Previous authors have voiced concern that coyotes would continue to be a persistent threat to red wolf recovery because they could occupy marginal habitat that red wolves could not [40,81]. However, these studies did not consider the potential benefits of transient red wolves on the persistence and maintenance of the red wolf population within the Recovery Area. Red wolves and coyotes display similar use of habitats in which red wolves require larger home-ranges because of larger body sizes. Transients of both species are excluded from red wolf territories and use similar edge habitats and road networks to bide in marginal habitats adjacent to wolf territories. During 1990–2005, when there were fewer coyotes and human-caused mortalities, red wolves typically took over vacant areas following breeder deaths [3,23]. However, since 2005, the coyote population and shooting deaths of red wolves has increased in the Recovery Area, resulting in a declining wolf population and increased coyote encroachment in vacant areas [3,23]. Local red wolf densities may now be too low to support enough transients to effectively recover lost territories and disrupt coyote encroachment. Because of few red wolves (≤100) in the Recovery Area [23,82], coyotes can exploit and defending these marginal patches between red wolf territories from other coyotes. The findings from our study suggest that if the red wolf population increases and saturates the Recovery Area, the available space for coyotes would diminish and the number of transient wolves frequenting marginal habitats would increase. In doing so, transient red wolves would likely disrupt coyote territories in marginal habitats while biding for opportunities to acquire territories and mates.
